# Who provides care in the last year of life? A description of care networks of community-dwelling older adults in the Netherlands

**DOI:** 10.1186/s12904-019-0425-6

**Published:** 2019-05-15

**Authors:** Femmy M. Bijnsdorp, H. Roeline W. Pasman, Anneke L. Francke, Natalie Evans, Carel F. W. Peeters, Marjolein I. Broese van Groenou

**Affiliations:** 1 Amsterdam UMC, Vrije Universiteit Amsterdam, Department of Public and Occupational Health, Amsterdam Public Health research institute, Expertise Center for Palliative Care, De Boelelaan, 1117 Amsterdam, Netherlands; 2 Nivel, Netherlands Institute for Health Services Research, Otterstraat, 118 Utrecht, The Netherlands; 3 Amsterdam UMC, Vrije Universiteit Amsterdam, Department of Epidemiology & Biostatistics, Amsterdam Public Health research institute, De Boelelaan, 1117 Amsterdam, Netherlands; 4 Vrije Universiteit Amsterdam, Department of Sociology, De Boelelaan, 1081 Amsterdam, The Netherlands; 5 Amsterdam UMC, Vrije Universiteit Amsterdam, Department of Public and Occupational Health, P.O. Box 7057, 1007 MB Amsterdam, Netherlands

**Keywords:** Home-based care networks, Older people, Informal home care, Formal home care

## Abstract

**Background:**

Home-based care networks differ in size and composition, but little is known about the characteristics of care networks for those nearing the end of their lives. This study aimed to identify different types of home-based care networks of community-dwelling older adults in the Netherlands and to assess the association between care network type and the health status and socio-demographic characteristics of care recipients.

**Methods/design:**

We used data from participants of the Longitudinal Aging Study Amsterdam (2001–2013) with chronic diseases or functional limitations who died within 12 months of their last interview and received home based personal and/or household care (*n* = 146). Latent Class Analysis was used to model distinct end-of-life care networks among this pooled cross-section of older people whose characteristics imply care needs. The Akaike information criterion was used to determine the optimal model. Associations between network type and care recipient characteristics were explored using conditional inference trees.

**Results:**

We identified four types of care networks; a *partner network* (19%) in which care was mainly provided by partners, with little care from private caregivers or professionals, a *mixed network* (25%) in which care was provided by a combination of children, professionals and/or other family members, a *private network* (15%) in which only privately paid care was provided, and a *professional network* (40%) in which care was mainly provided by publicly paid professionals, sometimes with additional care from family or privately paid caregivers. Care networks near the end of life showed similar characteristics to those identified for older people more generally, but care seemed to be more intensive in the last year of life compared to the years preceding it. End-of-life care networks were mostly related to age, educational level and partner status. Formal care substitutes informal care whenever there is no partner or child present and able to provide care.

**Conclusion:**

Our findings indicate that personal and household care can be quite intensive in the last year of life, especially for partner caregivers. To prevent caregiver burden, it is important that professionals make sure partner caregivers receive adequate and timely support to cope with the care situation.

**Electronic supplementary material:**

The online version of this article (10.1186/s12904-019-0425-6) contains supplementary material, which is available to authorized users.

## Background

For many, end-of-life care can be complex and intensive [1], dependent on the type, chronicity and severity of health problems. As most people prefer to remain at home as long as possible, and health care policies in many countries aim to substitute residential long-term care with community based care, care at the end of life is often provided at home [2, 3]. In general, care may be provided by a variety of caregivers: publicly paid professionals (e.g. home care staff), informal caregivers (e.g. relatives, friends), volunteers or privately paid caregivers [4]. These different types of caregivers together are described as the care network [5, 6], and this network may differ in size and composition. Various care network structures have been identified for community-dwelling older people, such as spousal/co-residential [4, 7], diverse informal [4], largely publicly paid [4, 8], and privately paid [4] networks. In spousal networks, few caregivers other than a partner are involved, while care in diverse informal networks is mostly provided by adult children and other informal caregivers [4, 7]. Publicly paid networks consist of formal caregivers and some informal caregivers [4, 8]. In privately paid networks there are hardly any other types of caregivers present, while the share of informal and formal caregivers is (more) equally divided in mixed care networks [4]. However, these studies did not focus on care received near the end of life. Previous work showed that informal care comprised a substantial proportion of the care received by older adults at the end of life [1, 9–11]. A Dutch study that compared care received by older adults in the last three months of life between 2000 and 2010, revealed a significant increase in the use of formal home care, or a combination of formal and informal home care in 2010 compared to 2000 [12]. Informal and formal care use near the end of life may even have further increased after 2010. However, the size and composition of care networks at the end of life remains unknown.

From research in the general population of community-dwelling older people, it is known that there are a number of individual characteristics associated with health service utilization, which can result in different types of care networks. Following the behavioral model of health care use, these characteristics can be divided in predisposing, enabling, and need factors [13]. Predisposing factors include demographic characteristics such as gender, age and education. Enabling factors include resources that facilitate the use of care, such as income and marital status [4, 13]. Need factors include health status and level of illness. People with certain individual characteristics are more likely to use health services than others [13]. For instance, being frail [14], being older (75+), having difficulties with instrumental activities of daily living or living alone are associated with more formal home care [9]. Disability level and serious chronic illness are associated with more formal and informal care use in the last life year, while living alone decreased the amount of informal care received [15]. Among older adults without disability, living alone or receiving informal care is also related to formal home care utilization [9]. Having a higher income is associated with the use of more privately paid and less publicly paid care, and the presence of a spouse is related to more informal care [4]. Among those living alone, frequent contact with friends, having children, and difficulties with instrumental activities of daily living are also related to receiving more informal care [16]. In general, women have larger and more diverse care networks than men, while men are more likely to receive family care from women as they have higher odds of being married in old age. Also, care networks become larger when morbidity increases, especially for women [17]. Formal care use among older adults near the end of life is related to being female, older in age (80+) or having lower functional ability [12].

To date, it is unclear which types of home-based care networks exist specifically near the end of life and how they are associated with characteristics of the care recipient. Knowledge about the types of networks at the end of life could help home care staff to recognize which end-of-life informal caregivers share care tasks with others and who provides care on their own. The latter may be in need for (more) professional support to relieve caregiver burden. In addition, detailed information about care networks at the end of life makes it possible to compare the size and composition of these networks to care networks in other life phases, and helps us understand whether and how care networks change closer to death. The aim of this study is to identify different types of home-based care networks of community-dwelling older adults in the Netherlands and to assess the association between different types of home-based care networks and the health status and socio-demographic characteristics of care recipients.

To better contextualize the findings, we first provide a brief overview of the Dutch healthcare system and the eligibility criteria of publically-funded home care support. In the Netherlands, professional personal care is funded by a public mandatory insurance, under the condition that a formal needs assessment has indicated that a person is in need of this care and informal caregivers are not able to provide this type of care. The general responsibility of the organization of household care is in the hands of municipalities. When people are not able to do household chores, due to (chronic) illness or disability, and informal caregivers are not able to provide this type of care, they can appeal for publically-funded home care support provided by their municipalities. As they have to pay a contribution for help from municipalities, some find it cheaper and easier to hire someone who can help with household chores themselves.

## Methods

### Study design and sample

The data were derived from the Longitudinal Aging Study Amsterdam (LASA), an ongoing longitudinal study of older adults in the Netherlands. This program consists of a nationally representative sample of older adults, stratified by age and gender, drawn from 11 municipalities that vary in religion and level of urbanization [18]. In 1992–1993, a total of 3107 participants born in 1903–1938 took part in the first LASA observation. Since then, data collection has taken place every three years. Additional samples of 55–64 years old participants were added to the study in 2002 and 2012. In following observations, these new cohorts were merged with the original cohort. More information about the sampling, data collection and cooperation rates can be found elsewhere [18, 19].

**Fig. 1:**
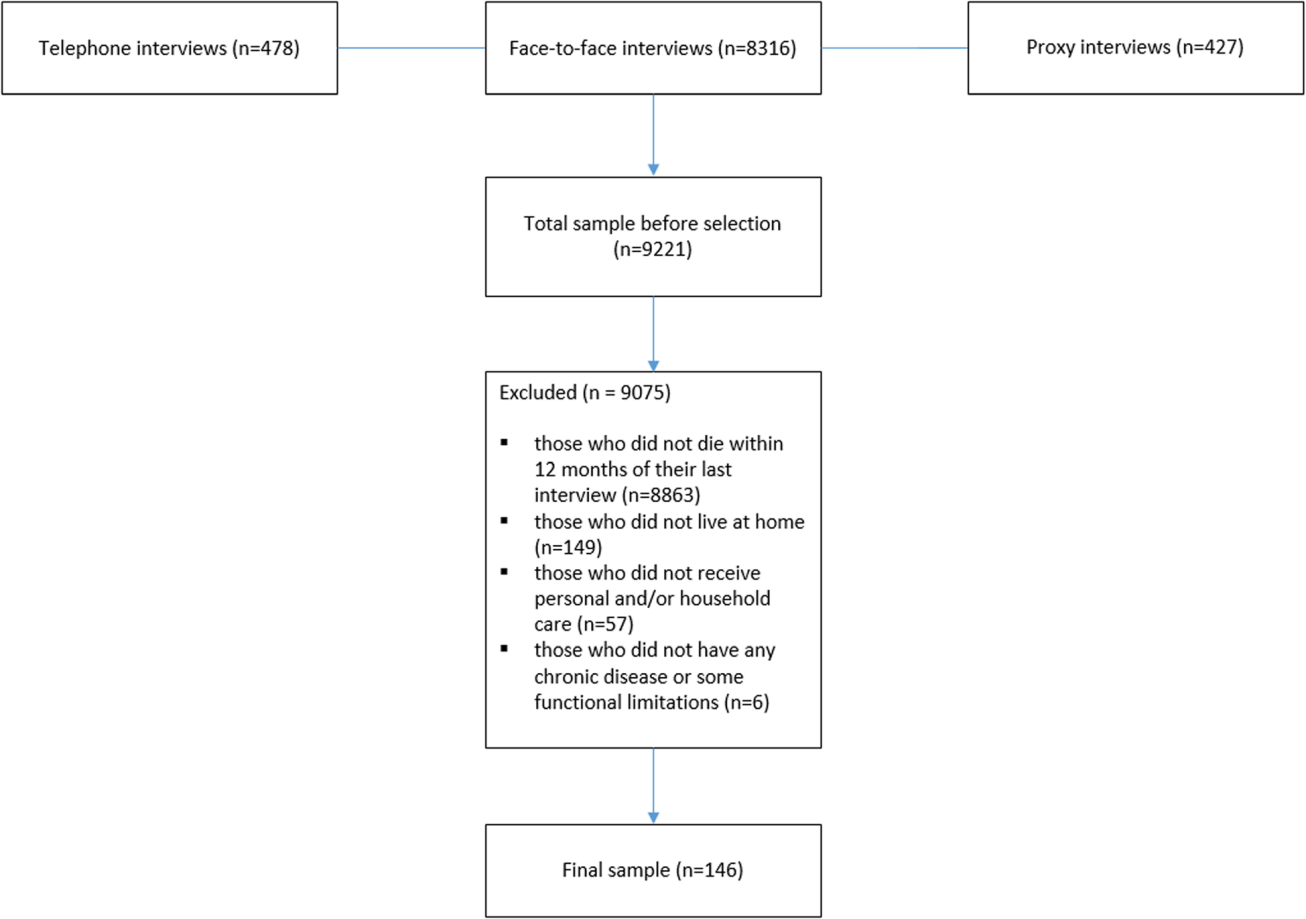
Flow chart of the selection procedure. *Notes*: All participants provided informed consent at baseline. From 2011/2012 onwards informed consent was provided before every new measurement. More information on the sampling, data collection, cooperation rates and reasons for drop-out per wave can be found in [18, 19]

For our study, we pooled the respondents who participated in the face-to-face interviews on the six observations between 2001 and 2013 (*n* = 8316). We also added the respondents who, due to health problems, only participated in a telephone interview (*n* = 478). When participants were too ill to participate themselves a proxy was interviewed (*n* = 427). Thus, the total sample before selection pooled from the six observations was *n* = 9221. We then excluded those who did not die within 12 months of their last interview (*n* = 8863), who did not live at home (*n* = 149), who did not receive personal and/or household care (*n* = 57) and those who did not have any chronic disease or some functional limitation (*n* = 6). In this way we selected a group of community-dwelling older people whom it could be expected to have care needs at the end of their lives. The final sample included 146 participants (see Fig. 1 and Additional file 1 for n-respondents per wave).

### Questionnaire

The questionnaire consisted of structured questions about socio-demographic characteristics, partner status, health (e.g., chronic diseases, functional capacity and cognitive functioning), use and type of care. *Chronic diseases* included cancer, chronic non-specific lung disease (CNSLD), heart disease, diabetes and stroke. For each disease type, participants reported whether the disease was present or not. The number of chronic diseases was used to indicate the presence of (co)morbidity. *Functional capacity* was measured with six questions about activities of daily living such as “Can you walk up and down the stairs?” [20]. The scale ranged from 0 = no difficulties to 6 = all with difficulty. A sum score was calculated by counting the number of items ‘with some difficulty’ or worse, with higher scores indicating more functional limitations. *Cognitive functioning* was based on the shortened version of the Mini-Mental State Examination (MMSE) [21] and consisted of 9 items. Scores ranged from 0 to 16, with higher scores indicating better cognitive functioning.

Participants were asked if they received personal care (e.g. bathing, toileting) and/or household care (e.g., cooking, cleaning) to determine the *use of care*. If answered affirmatively, participants were asked who provided the care and for how many hours per week. Care was given by informal caregivers (e.g. partner, relatives, friends), home care staff (e.g. home care nurses, auxiliary nurses), privately paid care (no, yes) and volunteers (no, yes). However, none of the older adults in the last year of life received care from volunteers. Care from home care staff and privately paid caregivers in this study consisted of practical care that could also be given by informal caregivers (personal and/or household care) and did not include medical and nursing care.

### Analyses

#### Identifying care networks

**Fig. 2:**
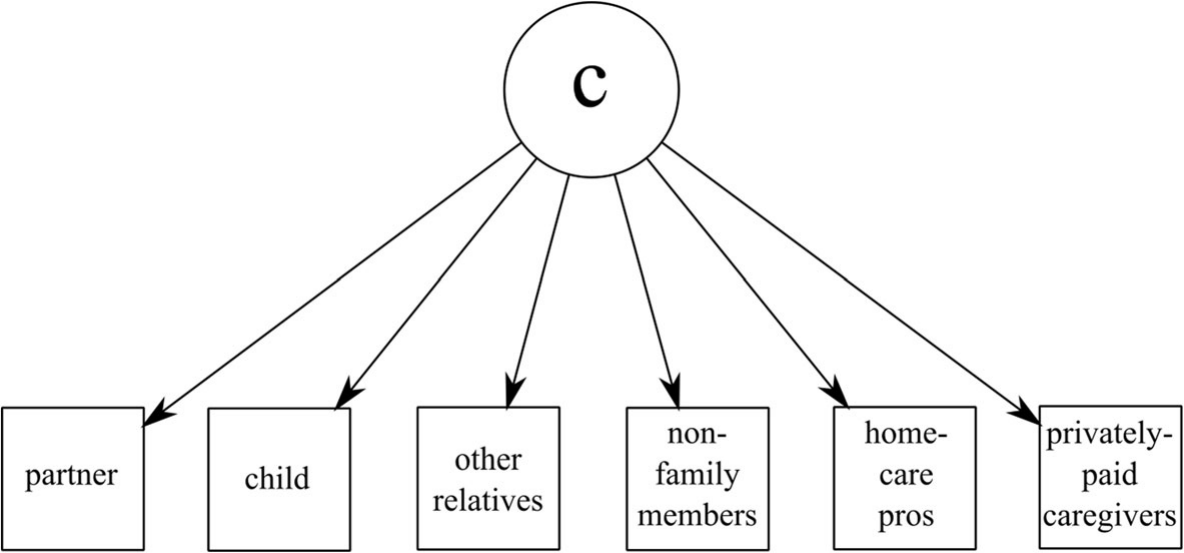
Path diagram of the LCA model on our 6 manifest variables. *Notes:* The manifest variables (in boxes) are assumed to be indicators for the underlying categorical latent variable *c* whose nominal categories are referred to as latent classes

Latent class analysis (LCA) was employed to identify types of care networks among older adults in the last year of life. LCA is based on a latent variable model that relates manifest categorical variables to a categorical latent variable whose nominal categories are referred to as latent classes [22]. The aim was to identify the latent classes (interpreted as care network types) amongst binary variables (yes/no) indicating if one received care from: (1) a partner, (2) a child, (3) other relatives, (4) non-family members, (5) home care staff, (6) privately paid caregivers. A path diagram of the LCA model with these manifest variables is given in Fig. 2. The analysis made use of the poLCA package [23, 24] in R [25]. This package fits the LCA model by the expectation-maximization algorithm. To avoid local maxima each model (conditional on the latent dimensionality) is refitted 100 times using random starting-values. The fit corresponding to the greatest log-likelihood is retained. This procedure is repeated for models with 2 to 5 latent classes. The optimal number of latent classes is then determined by assessing the Akaike information criterion (AIC), which balances model fit with model complexity [26]. The model with the lowest AIC is deemed optimal (given that the solution is interpretable). When the analysis stresses the identification of all relevant classes and when indeed a complex solution is expected (in terms of a relatively high number of latent classes) the AIC is to be preferred over other possible information criteria [27].

Class membership for the individuals in the study is then based on the final model. Posterior class membership probabilities are calculated and each individual is assigned to the latent class on which this probability is the highest [24]. The fit of the final model is assessed by looking at the concordance between the estimated mixing proportions and the population share belonging to each latent class based on the class-assignment according to the posterior model probabilities: Congruence is indicative of a good fit [24]. Moreover, it was assessed if the manifest variables indeed discriminate between the retained latent classes by evaluating the association between them. This evaluation is based on a chi-square test with a Monte-Carlo-based *p*-value (50,000 permutations) followed by Bonferroni correction (see Additional file 2 for model fit evaluation and latent class solution).

#### Characterizing care networks

First, we described the care networks based on the descriptive statistics of the care recipient and care situation. After that, we explored associations between network type and care recipient characteristics using conditional inference trees (CITs). CITs are non-parametric regression trees. They embed tree-structured regression models into a recursive conditional inference procedure [28]. Our analysis made use of the party package [28] in R. A quadratic-type test statistic was applied for variable selection. The stopping criterion was based on Bonferroni adjusted Monte-Carlo *p*-values (50,000 permutations). As this analysis was exploratory in nature, we set the nominal level of the conditional independence tests at 0.1. The tree was grown with the minimum parent node-size for splitting set at 20 and minimum terminal node-size set at 5. Variables considered in the CIT were age (continuous), sex (binary), educational level (ordinal with categories ‘low’, ‘middle’, and ‘high’), and number of chronic conditions (ordinal with categories ‘0’, ‘1’, and ‘2 or more’).

## Results

### General characteristics of the sample

The characteristics of the general sample are set out in Table 2. About half were male (52.1%) and the average age was 81 years old. More than half of the sample had a low level of education (57.5%), followed by medium-level of education (27.4%) and high level of education (15.1%). Less than half of the older adults had a partner (45.4%). On average, respondents had 1.2 chronic diseases, with cancer being the most prevalent (36.3%), followed by heart disease (31.5%), CNSLD (23.3%), stroke (16.4%) and diabetes (14.4%). Older adults had quite a few functional limitations, but relatively good cognitive functioning.

### Care networks in the last year of life

**Fig. 3:**
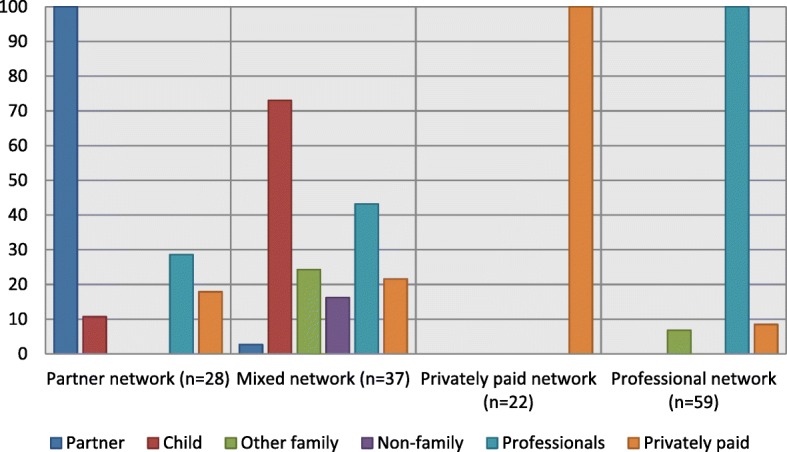
Type of caregivers in the last year of life (%). *Notes*: Classification based on posterior modal probabilities. The bars represent the percentage of affirmative answers on receiving care from corresponding colour-type caregiver

The LCA resulted in four distinct care-network types (see Fig. 3). The first network type is the *partner network (19%).* In this network, older adults in the last life year received care from their partner, sometimes supplemented with care from children, home care staff and privately paid caregivers (see Table 1). On average they received the most hours (about 30 on average) of care per week. The majority received household care from their partner and about half also received personal care. More than three-quarters of the care recipients were male, relatively young, and higher educated (see Table 2). Almost half of them suffered from cancer and one-third from a heart disease. Functional limitations were a little below average (3.3 versus 3.9 on average).

**Table 1:** Descriptive statistics of the living situation per care-network type

	Total sample	Partner network (*n* = 28)	Mixed network (*n* = 37)	Privately paid network (*n* = 22)	Professional network (*n* = 59)
*Characteristic*	*M (SD)*				
Total average hours of care ^a^ (informal/formal) (1–177)	13.5 (31.6)	29.9 (52.5)	16.4 (38.1)	3 (1.7)	7.8 (9.9)
Received informal care with personal care					
Partner (%)	9.6	46.4	2.7	0	0
Hours of care from partner (0–168)	2.6 (19.6)	13.8 (43.7)	0	0	0
Child (%)	2.7	0	10.8	0	0
Hours of care from child (0–30)	0.3 (2.7)	0	1.2 (5.4)	0	0
Other family (%)	2.7	0	5.4	0	3.4
Hours of care from family (0–21)	0.2 (1.7)	0	0.1 (0.4)	0	0.4 (2.7)
Other non-kin (%)	0	0	0	0	0
Hours of care from non-kin (0)	0	0	0	0	0
Received informal care with household tasks					
Partner (%)	15.8	78.6	2.7	0	0
Hours of care from partner (0–168)	2.8 (15)	14.3 (32.3)	0.2 (1.2)	0	0.1 (0.5)
Child (%)	19.9	10.7	70.3	0	0
Hours of care from child (0–168)	1.7 (14)	0.3 (0.8)	6.6 (27.4)	0	0
Other family (%)	7.5	0	21.6	0	5.1
Hours of care from family (0–168)	1.4 (14)	0	4.8 (27.6)	0	0.5 (2.8)
Other non-family (%)	4.1	0	16.2	0	0
Hours of care from non-family (0–1)	0 (0.2)	0	0.2 (0.4)	0	0
Received professional care with personal care					
Professional home care (%)	31.5	14.3	27	0	54.2
Hours of professional home care (0–42)	2.1 (6.2)	0.5 (1.7)	1.7 (4.7)	0	4 (8.5)
Privately paid care (%)	0	0	0	0	0
Hours of privately paid care (0)	0	0	0	0	0
Received professional care with household tasks					
Professional home care (%)	45.9	21.4	29.7	0	84.7
Hours of professional home care (0–6)	1.3 (1.8)	0.5 (1.2)	0.7 (1.5)	0	2.6 (2)
Privately paid care (%)	27.4	17.9	21.6	100	8.5
Hours of privately paid care (0–7)	0.9 (1.7)	0.5 (1.2)	0.8 (1.7)	3 (1.7)	0.3 (1.2)

Notes: Standard deviations are given in parentheses. ^a^ Hours of care per week

**Table 2:** Characteristics of the care receiver and bivariate associations with care-network type

	Total sample	Partner network 1 (*n* = 28)	Mixed network 2 (*n* = 37)	Privately paid network 3 (*n* = 22)	Professional network 4 (*n* = 59)	Statistic (χ2/F)	*p*-value	Post-hoc comparison of means/Pairwise comparison
*Characteristic*	*M (SD)*							
Gender (% female)	47.9	21.4	62.2	45.5	52.5	11.4	.010	
Age (55–95)	81.3 (9.2)	76.6 (9.5)	83.0 (8.9)	79.8 (9.3)	83.2 (8.6)	4.0	.009	1 < 2,4
Educational level (%)						16.8	.010	
Low	57.5	50	70.3	27.3	64.4			
Middle	27.4	25	24.3	54.5	20.3			
High	15.1	25	5.4	18.2	15.3			
Partner (%)	45.4	100	13.5	45.5	39	49.9	.000	
*Nature of health problems*								
Chronic diseases (0–5)	1.2 (1.2)	1.4 (1)	1 (1)	1.1 (1)	1.3 (0.2)	0.6	.625	
CNSLD (%)	23.3	14.3	18.9	27.3	28.8	2.9	.412	
Heart disease (%)	31.5	32.1	27	40.9	30.5	1.3	.734	
Diabetes (%)	14.4	21.4	13.5	9.1	13.6	1.7	.640	
Stroke (%)	16.4	21.4	18.9	4.5	16.9	3	.399	
Cancer (%)	36.3	46.4	24.3	31.8	40.7	4.2	.239	
Functional limitations (0–6)	3.9 (2)	3.3 (2.1)	4.4 (1.9)	3 (1.9)	4.1 (1.9)	11.3	.010	2 > 3
Cognitive functioning (1–16)	13.8 (2.7)	13.3 (2.5)	13.6 (3)	14.7 (1.5)	13.3 (3)	6.8	.078	

Notes: Standard deviations are given in parentheses. The reported p-values are raw p-values (without multiplicity correction)

Second, a *mixed care-network (25%)* was identified in which older adults in the last year of life mainly received care from children, sometimes supplemented with care from home care staff, other family members, non-family members, privately paid caregivers and, in just a few cases, partners. This group received about sixteen hours of care per week on average. They received relatively more household and personal care from children, other family members and non-family members. Children and family members also provided the most hours of care, mainly with household tasks. The majority (62%) of the care recipients were women, who were relatively older (average age is 83), lower educated, and with more functional limitations. They rarely lived with a partner (14%) and, when they did, partners provided very little care, possibly because they were also old and frail.

Third, a *privately paid network (15%)* was distinguished. Older adults received in the last year of life household care from privately paid caregivers, on average three hours per week, and no care from any other type of caregiver. About 40% suffered from heart failure, one-third (32%) from cancer, and one-quarter (27%) from lung disease, yet they had the least functional limitations and highest cognitive functioning on average. Overall, their health was better than in other networks, which could explain the absence of any informal and professional caregiver.

Fourth, a *professional network (40%)* was identified in which the older persons received care from home care staff. Older adults received eight hours of care per week on average. Professionals mainly provided household care and in more than half of the cases they also provided personal care. Care from professionals was, in a few cases, supplemented with personal and household care from other family members, and household care from privately paid caregivers. Care recipients in this network were relatively old, about one-third lived with a partner, and a large proportion (64%) had a low level of education. Relatively more older adults suffered from cancer (41%), heart failure (31%) lung disease (29%), and functional limitations were a little above average (4.1 versus 3.9 on average).

### Determinants of care networks

**Fig. 4:**
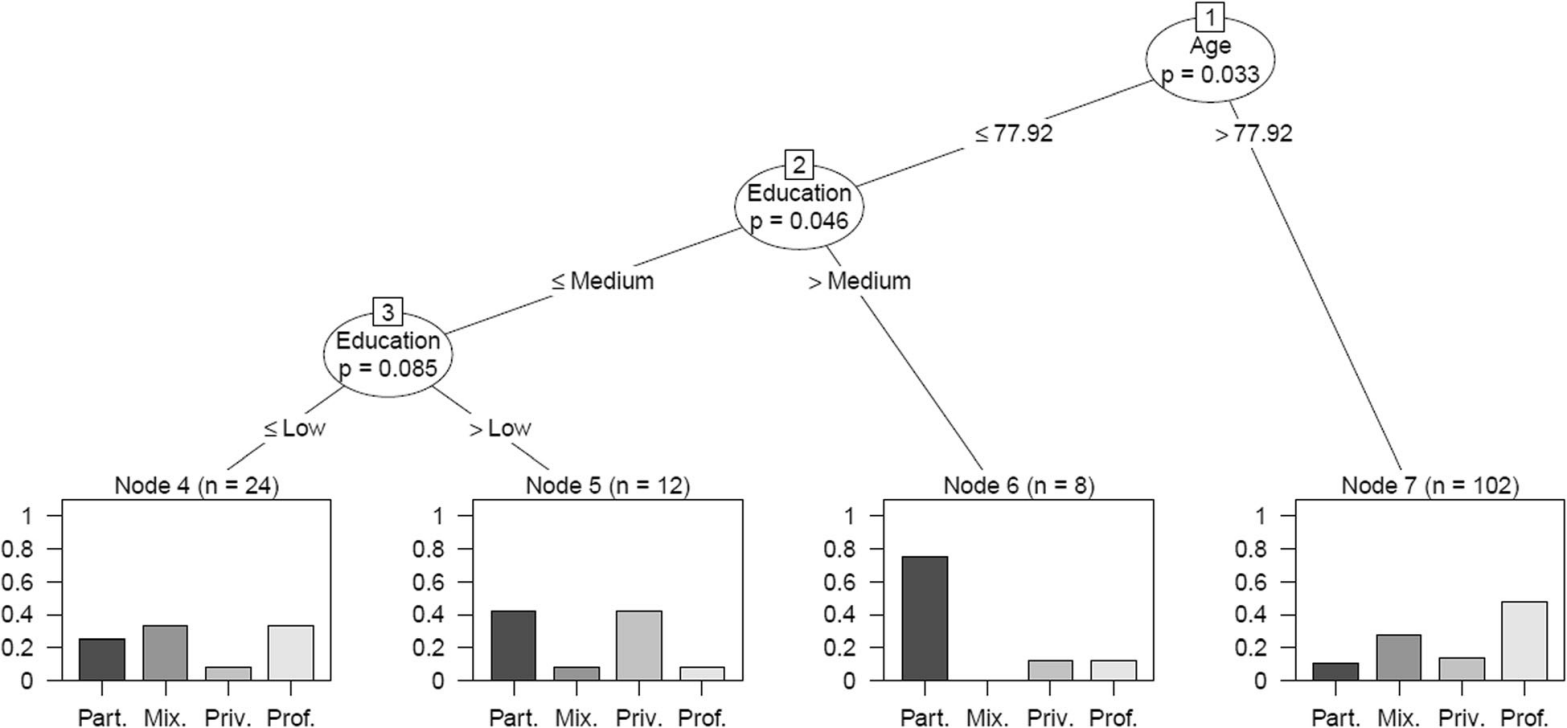
Conditional Inference Tree for Class. *Notes:* The root node is split according to age with 77.92 years forming the optimal binary-split cut-off. The lower age-category (those with ages equal to or lower than 77.92 years of age) can be further subdivided according to education. It is split into a higher educational attainment node (> Medium) and a medium to lower educational attainment node (≤ Medium). Lastly, the node reflecting the younger age group with ≤ Medium education is further split, again according to educational attainment, into a lower educational attainment node (≤ Low) and a medium educational attainment node (> Low). Further interpretation can be found in the text. Abbreviations; Part. = partner network, Mix. = Mixed network, Priv. = Privately paid network and Prof. = Professional network

To explore which socio-demographic characteristics of the care recipient might be associated with network type, we extracted a CIT. In our CIT (see Fig. 4) the covariate showing the largest association to type of care-network is age. Two age groups were distinguished; younger than 77.9 years and above 77.9 years of age. The type of network in the older age-group is predominantly mixed and often involves professionals. The lower age-category can be subdivided according to educational level. For those in the lower age-category, higher educational attainment identifies persons that predominantly have a partner network. For those in the lower age-category, a medium level of education is associated with partner and privately paid networks, while low level of education is associated with professional and mixed networks.

## Discussion

This study identified four different types of home-based care networks among community-dwelling older adults in the last life year in the Netherlands: the partner network, the mixed network, the privately paid network and the professional network. Our study shows that the majority of older adults in the last year of life have various types of caregivers in their network, and that there is also variation in this mix because we have differentiated between types of informal caregivers (partners, children, other relatives and non-family). This is in line with Kemp and colleagues [6] who argue that the formal-informal care intersection is more complex than the ‘simplistic’ models of informal care only, formal care only or a mix of informal and formal care. The role of informal and formal caregivers varied between the networks and the composition of the care networks was mostly related to age and educational level. Similar to previous findings [9, 12], professional home care staff was predominantly involved in the care of the oldest-old (aged over 78). Nearly 40% of the care recipients in the professional network had a partner, but did not receive care from them. It could be that this spouse was also old and frail. Care recipients in the partner network received little formal care, those in mixed care networks (care from child, no partner) more often received formal care, and those in professional care networks (no care from partner or children) received relatively more hours of formal care. This indicates that formal care substitutes informal care whenever there is no partner or child present and able to provide care. Our findings corroborate that in particular women and the lower educated more often depend on formal care compared to men and the higher educated, due to a lack of informal care [29]. Our results may particularly apply to community-dwelling older adults in the Netherlands or to older adults in countries with similar policies towards care provision, because the role of informal and formal caregivers is often found to depend on country specific policies [30, 31]. Type of disease proved not to be important in the distinction of care network types. It could be that the specified diseases did not require specific help regarding personal and household care in this population, as all care receivers were relatively old and required care.

### Care networks near the end of life compared to other life phases

The care networks near the end of life showed similar characteristics to those identified for older people more generally in other samples [4, 7, 8] with respect to the composition of involved caregivers. However, the intensity of care in the year before death seems to be higher. In other samples, a co-residential network was identified [4] in which older adults received about nine hours of care per week, which is much lower than the 30 h of care per week provided in our partner network. In an earlier identified large informal network [4] older adults received approximately five hours of care per week, while older adults in our mixed network type received up to sixteen hours per week. Another recent study even found that informal caregivers reported about 69 median hours of care for a relative in the last three months of life [32]. Hence, care can become quite intensive near the end of life and caregivers support needs are particularly high at this time [33].

### Implications for informal caregivers and professionals

Due to population ageing and the policy to shift towards more community based care [2, 3], it can be expected that informal caregivers will increasingly be involved in the care for relatives near the end of life. This has a number of implications for both informal caregivers and professionals. Providing care at the end of life might be particularly burdensome when care becomes intensive and support is lacking [33]. Partner caregivers provided the most intensive care and received little help from others. Hence, these caregivers might be at risk of becoming overburdened and deserve more support. This supports the idea that a shift towards collective community caregiving, in which both formal and informal caregivers share the caring work, is needed to avoid isolation and reduce the burden on primary informal caregivers [34].

From the perspective of the professional, it is important to make sure caregivers receive adequate and timely support to cope with the care situation, in order to prevent caregiver burden. However, it is also known that some informal caregivers, in particular spouses, can be quite reluctant to involve other caregivers and hold back from asking for help [35]. In these cases, professionals could take the initiative and explore the possibilities to involve other informal caregivers or volunteers in order to prevent or decrease burden. This could also facilitate ‘collective community caregiving’, by initiating and maintaining such informal networks and placing caring networks as central to the caregiving process [34]. Also, professionals could provide tailored support by assessing the specific needs of the informal caregivers involved [36, 37]. Special attention is needed for partner caregivers, but these ‘needs assessments’ are also useful for other types of informal caregivers. There are several tools that professionals could use to screen for needs of relatives. One of these tools is the Caregiver Strain Index, measuring level of strain [38]. Another more recently developed tool is the Carer Support Needs Assessment Tool (CSNAT) [39]. This is an evidence based tool for the direct measurement of carers’ support needs on several domains and is specifically developed for end-of-life care.

### Strengths and limitations

To the authors’ knowledge, this is one of the first studies that identifies the composition of home-based care networks near the end of life and their association with characteristics of the care recipient. The advantage of our longitudinal cohort study is that information on mortality was available through linkage with registers of the municipalities in which the respondents were living. Therefore, we were able to identify which respondents died within 12 months of their last interview and had detailed information on their characteristics including health status and care use. However, at the time of measurement, it was not clear if older adults were aware that they were close to death or whether they were in the palliative phase. Although we only selected data from people who had a severe disease or who were disabled, it is nearly impossible to measure if and when someone reaches the palliative phase in a longitudinal study with data collection only every three years. In addition, although we had information about the presence of a number of severe diseases that are associated with mortality, information about degenerative neurological diseases was not available. We used the level of cognitive functioning of the care receivers as a proxy for neurological diseases, and respondents with a low level of cognitive functioning did not participate in our face-to-face interview. Although we added observations from the telephone and proxy interviews (from participants with severe health problems), we don’t know if they could not participate in the face-to-face interview due to degenerative neurological diseases. This could explain the relatively good cognitive functioning of our study sample. In a sample including patients with degenerative neurological diseases, the proportion of mixed and professional networks may have been higher. Another limitation is that information on how many caregivers were involved in the networks was not available for everyone (only for those in the 2011–2012 wave). Therefore, care network size remains unclear and it is thus unknown by how many different caregivers tasks were shared and whether or not there are more people involved near the end of life compared to other times. In addition, none of the older adults in our sample received care from volunteers in the last year of life. Therefore, we were unable to include this type of caregiver in the construction of care networks at the end of life. Also, formal care did not include medical and nursing care. Hence, the composition of care networks might differ when these types of care and/or volunteers are also included.

Further, the characterization of the care networks by CITs might be subject to issues of confounding. First, it is possible that we did not include important auxiliary information in building such trees. Second, the best characterization of class-membership need not be tree-structured. Given our limited sample size our approach was necessarily exploratory. We aim to deal with these issues in potential follow-up research, using specialized methodology for confounder-adjustment [40, 41]. In addition, questions regarding the number of caregivers per caregiver type and information about the use of medical and nursing care could be included in future research to further assess this. The role of socio-economic characteristics of care recipients near the end of life, such as income, control beliefs and the social network could also be further explored.

## Conclusion

Care networks near the end of life were mostly related to age and educational level, and not to type of disease. Formal care substitutes informal care whenever there is no partner or child present and able to provide care. Our findings indicate that care can be quite intensive in the last year of life, especially for partner caregivers. More support is needed for informal caregivers is needed to prevent them becoming overburdened. Education about the network types that exist near the end of life could help professionals to provide tailored support. More research about the informal caregivers’ experiences of support in the care networks is needed to provide insight into their support needs.

## Additional files

Additional file 1: The numbers of respondents per wave in the final sample. (DOCX 12 kb)

Additional file 2: Latent class solution. (DOCX 14 kb)

## Abbreviations

AIC: Aikaike information criterion
CIT: Conditional inference tree
CNSLD: Chronic non-specific lung disease
CSNAT: Carer Support Needs Assessment Tool
LASA: Longitudinal Aging Study Amsterdam
LCA: Latent class analysis
MMSE: Mini-Mental State Examination
