# Supplementary material for: Who provides care in the last year of life? A description of care networks of community-dwelling older adults in the Netherlands
Source: BMC Palliat Care. 2019 May 15;18:41. doi: 10.1186/s12904-019-0425-6 (PMC6521417; doi:10.1186/s12904-019-0425-6)
Supplement: Supplementary file 1 — The numbers of respondents per wave in the final sample. (DOCX 12 kb) [file 12904_2019_425_MOESM1_ESM.docx]

**Additional file 1**

***The numbers of respondents per wave in the final sample***

| Table 4. Number of participants per wave | |
| --- | --- |
|  | n |
| 2001-2002 | 36 |
| 2002-2003 | 5 |
| 2005-2006 | 43 |
| 2008-2009 | 31 |
| 2011-2012 | 19 |
| 2012-2013 | 12 |
