# Supplementary material for: Who provides care in the last year of life? A description of care networks of community-dwelling older adults in the Netherlands
Source: BMC Palliat Care. 2019 May 15;18:41. doi: 10.1186/s12904-019-0425-6 (PMC6521417; doi:10.1186/s12904-019-0425-6)
Supplement: Supplementary file 2 — Latent class solution. (DOCX 14 kb) [file 12904_2019_425_MOESM2_ESM.docx]

**Additional file 2**
***Latent class solution***
Table 1 contains, for each class-solution, the model-fit information corresponding to the greatest log-likelihood over the refits. The 4-class model best balances model fit and model complexity according to the AIC. Moreover, each manifest variable appears to have discriminatory power when associated with the latent classes under the 4-class model (Table 2). In addition, the mixing proportions (estimated class population shares) are congruent with the predicted class memberships (Table 3). Most importantly, it gives interpretable latent classes (Figure 1, Main Text). Hence, the 4-class solution is retained as the final model.

**Table 1. Model fit evaluation**

| No. of latent classes | LL | Par. | AIC |
| --- | --- | --- | --- |
| 2 | -384.223 | 13 | 794.446 |
| 3 | -371.359 | 20 | 782.717 |
| 4 | -362.860 | 27 | 779.720 |
| 5 | -357.253 | 34 | 782.505 |

*Notes*: LL = maximum of log-likelihood,

Par. = number of estimated parameters,

AIC = Akaike Information Criterion.

**Table 2. Association between manifest variables and latent classes**

| Manifest variable | Chi-square value | *p*-value | Adjusted *p*-value |
| --- | --- | --- | --- |
| Partner | 139.89 | $2\times{10}^{-5}$ | $.00012$ |
| Child | 84.90 | $2\times{10}^{-5}$ | $.00012$ |
| Other relatives | 16.06 | $.00144$ | $.00864$ |
| Non-family members | 18.43 | $.00048$ | $.00288$ |
| Home care professionals | 85.69 | $2\times{10}^{-5}$ | $.00012$ |
| Privately-paid caregivers | 70.82 | $2\times{10}^{-5}$ | $.00012$ |

**Table 3. Mixing proportions and predicted class memberships**

|  | Class 1 | Class 2 | Class 3 | Class 4 |
| --- | --- | --- | --- | --- |
| Mixing proportions | .166 | .312 | .166 | .357 |
| Predicted class memberships | .192 | .253 | .151 | .404 |
